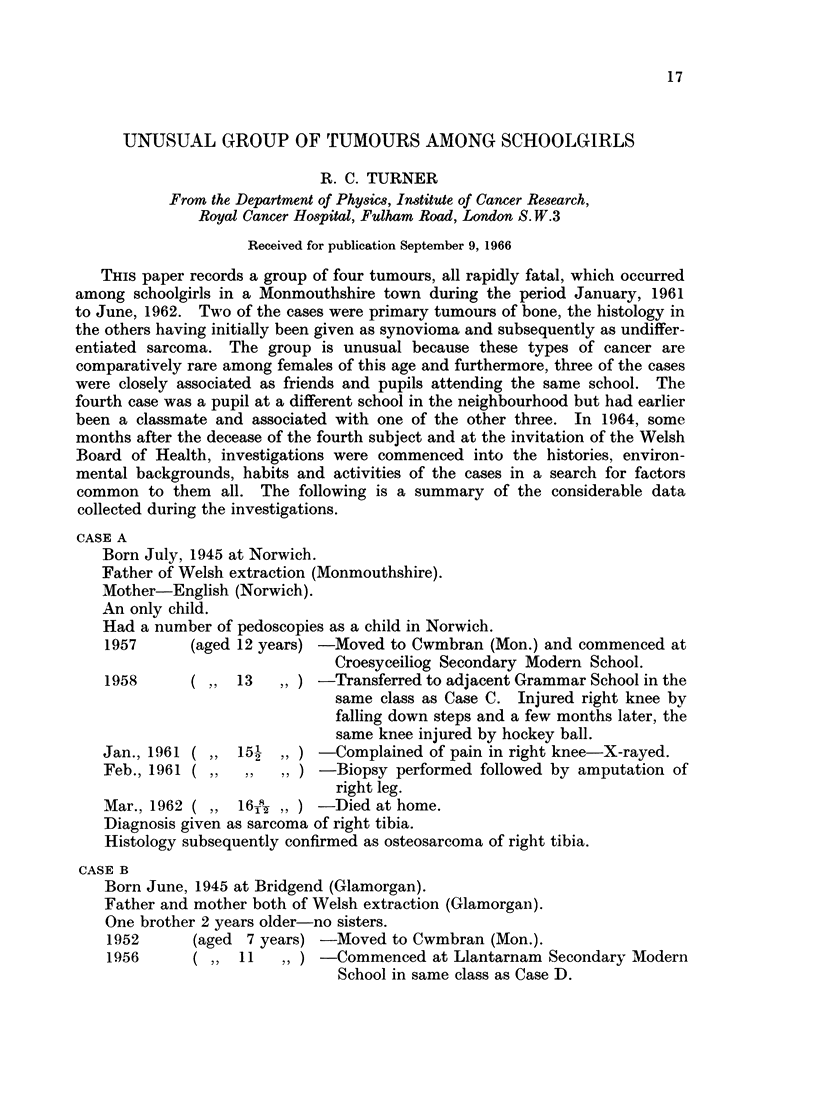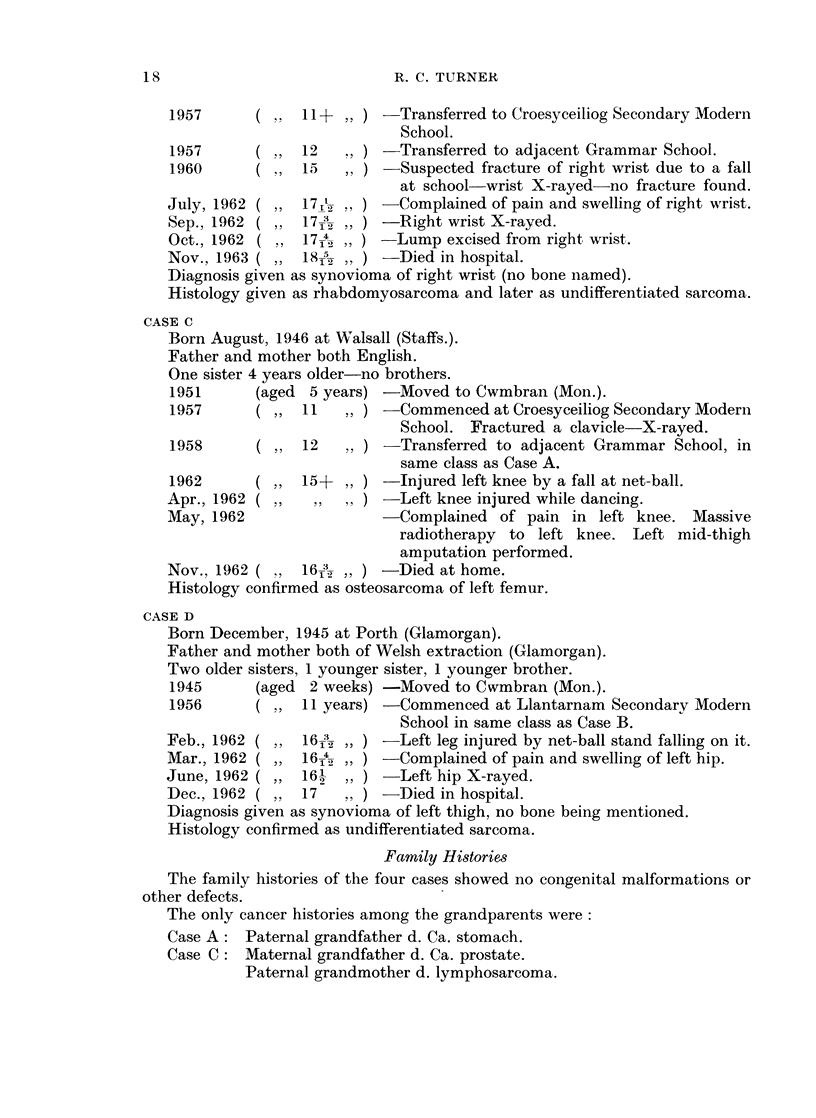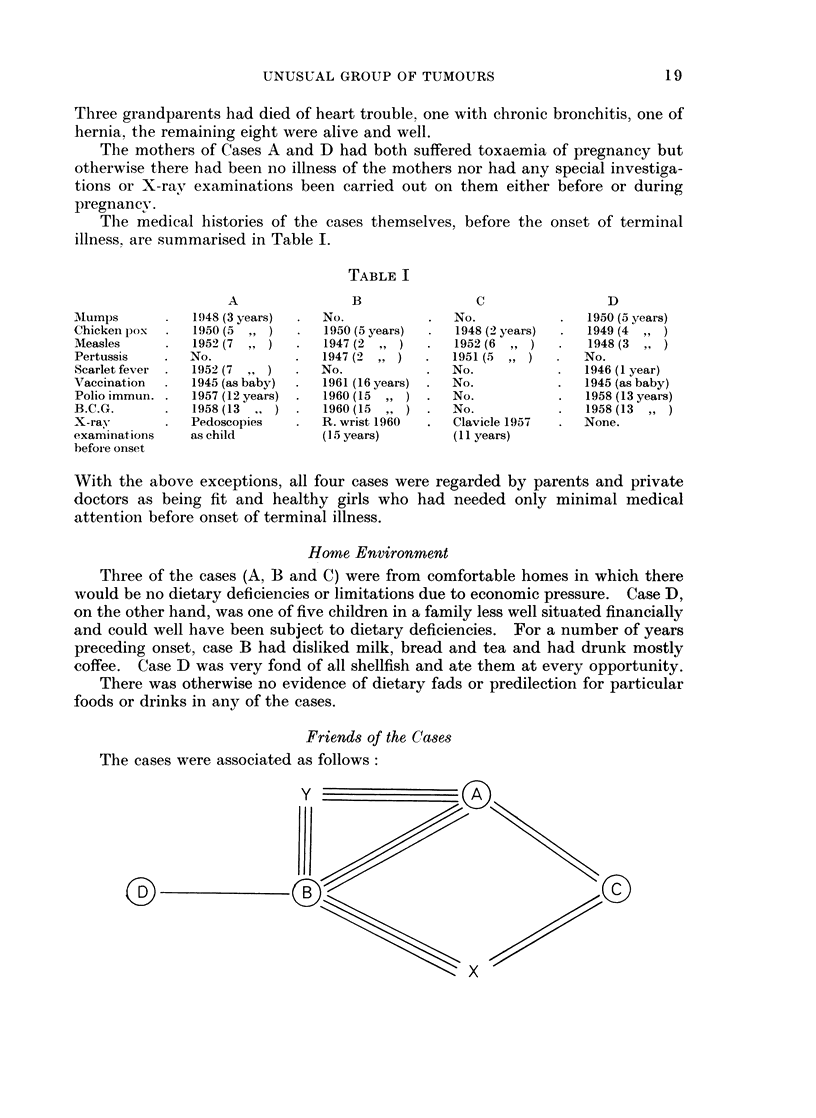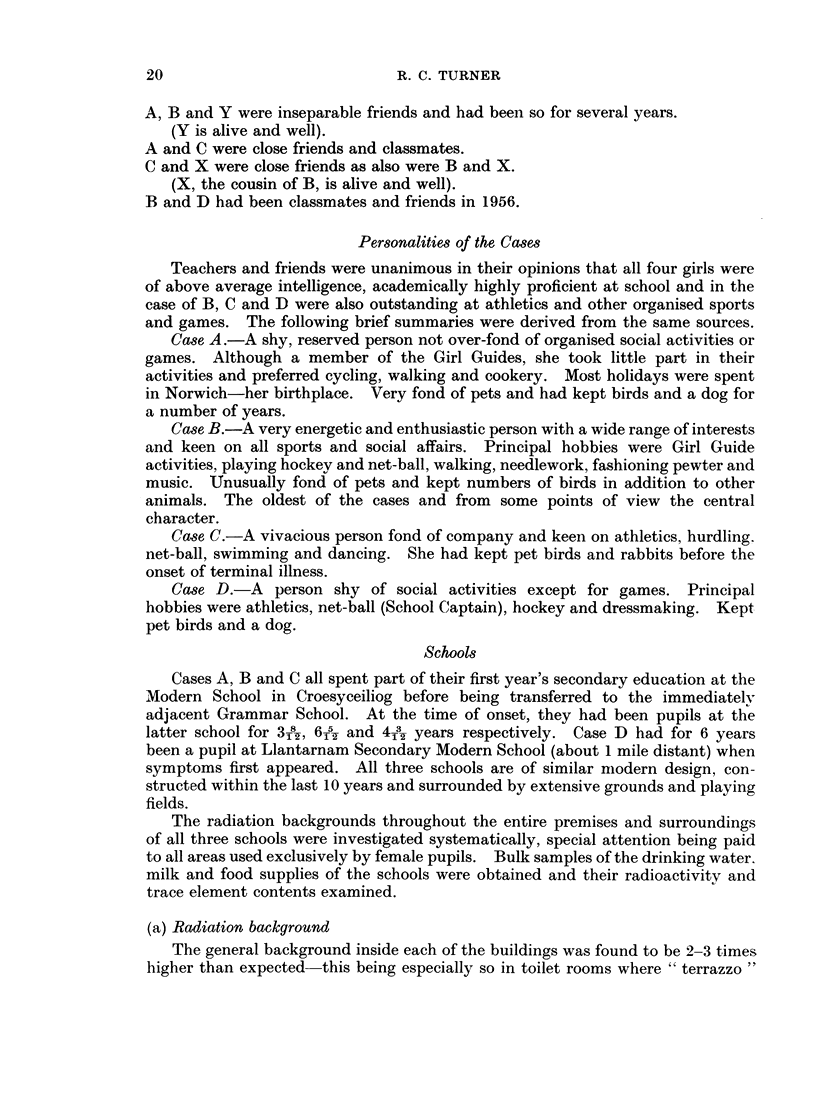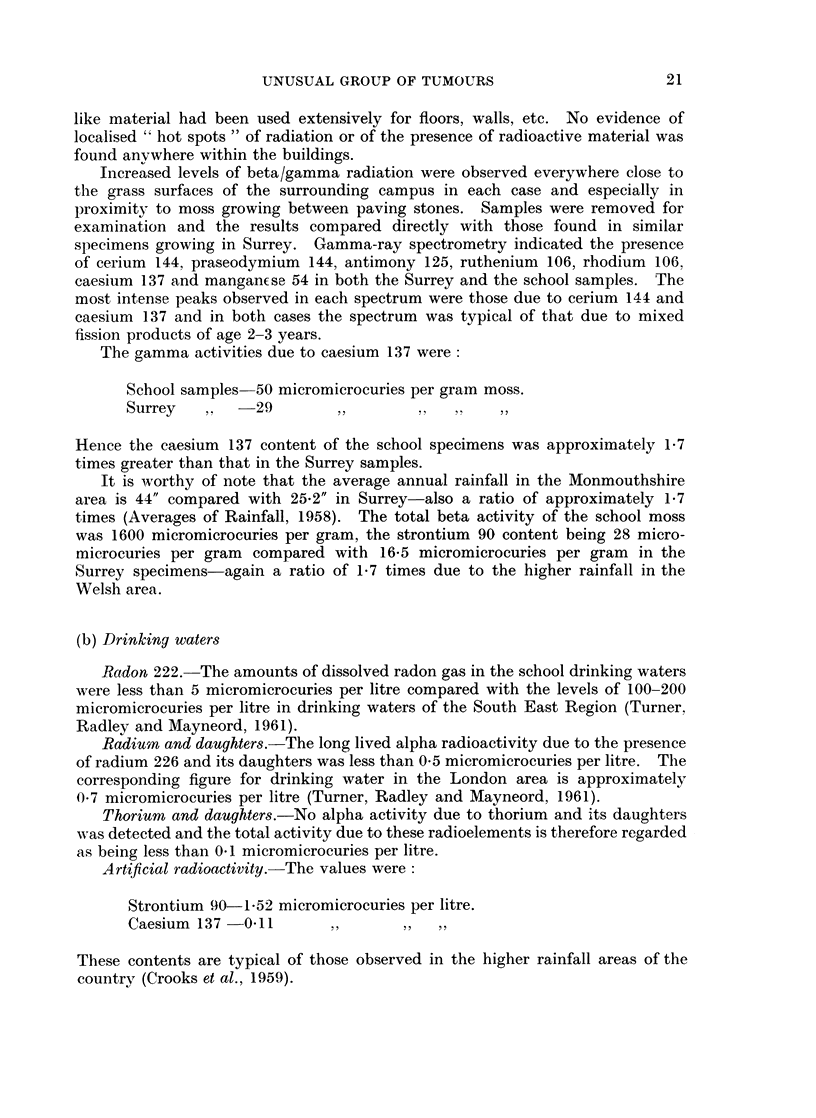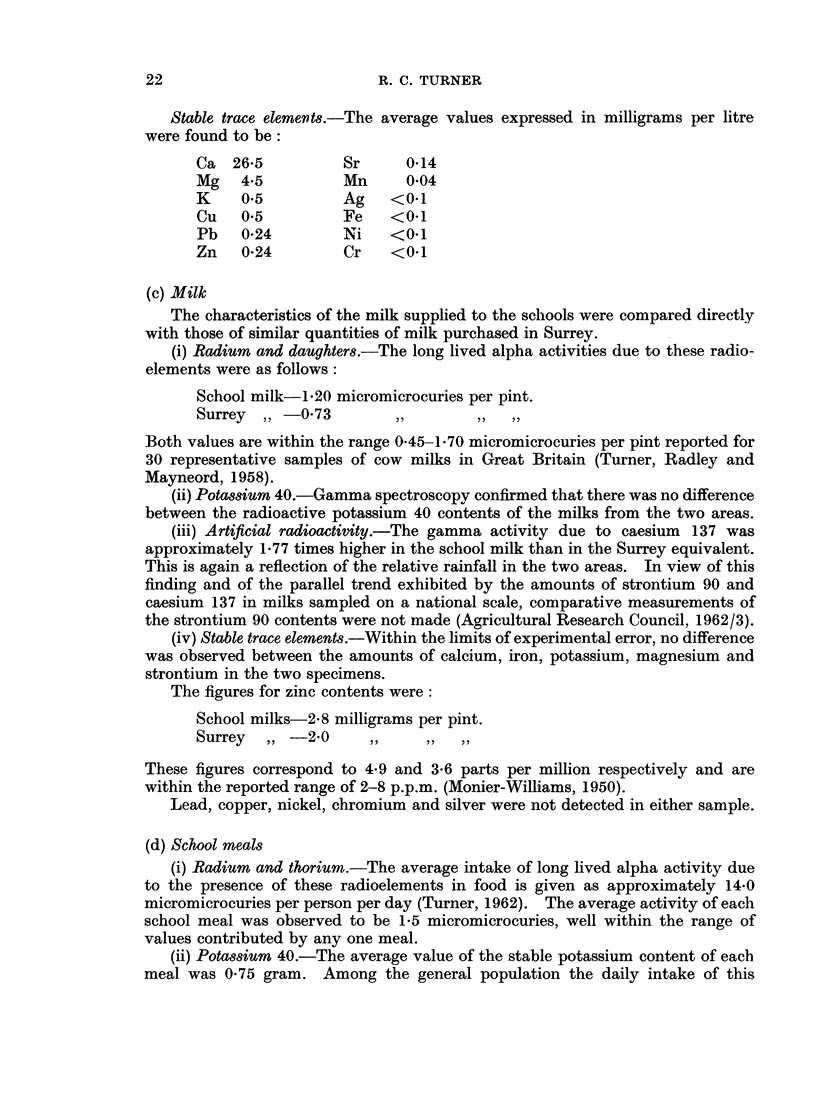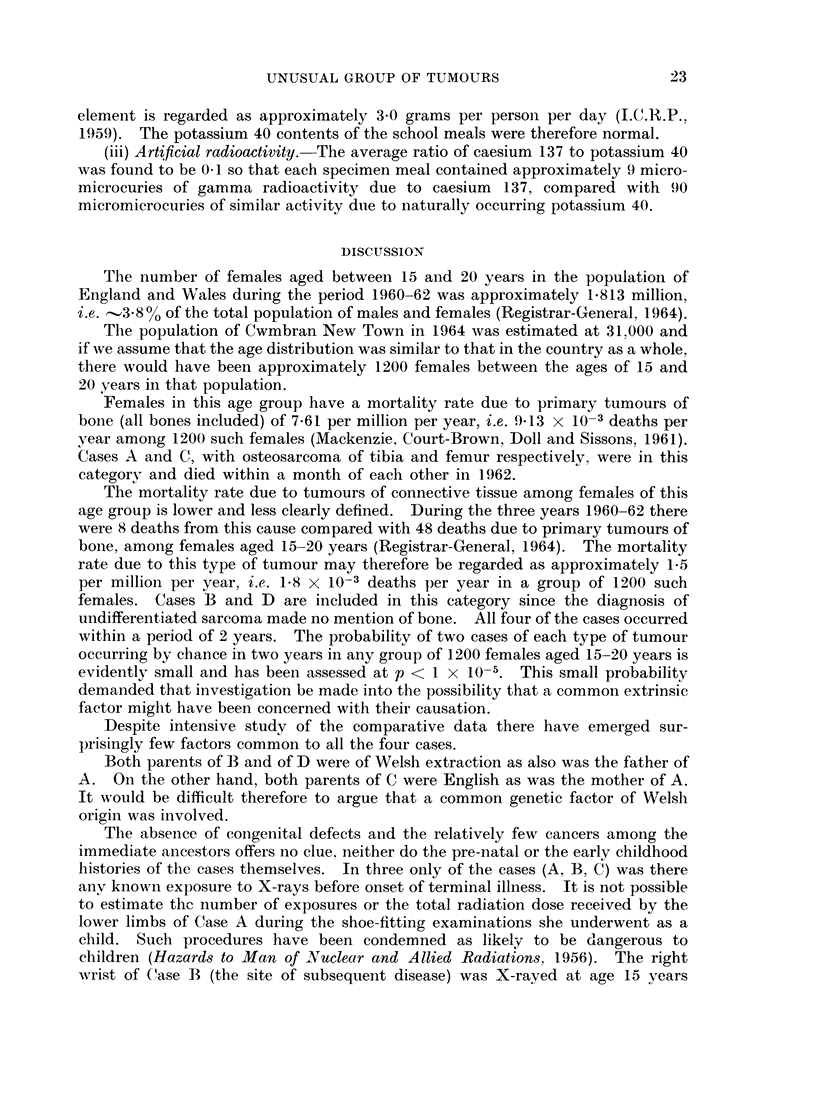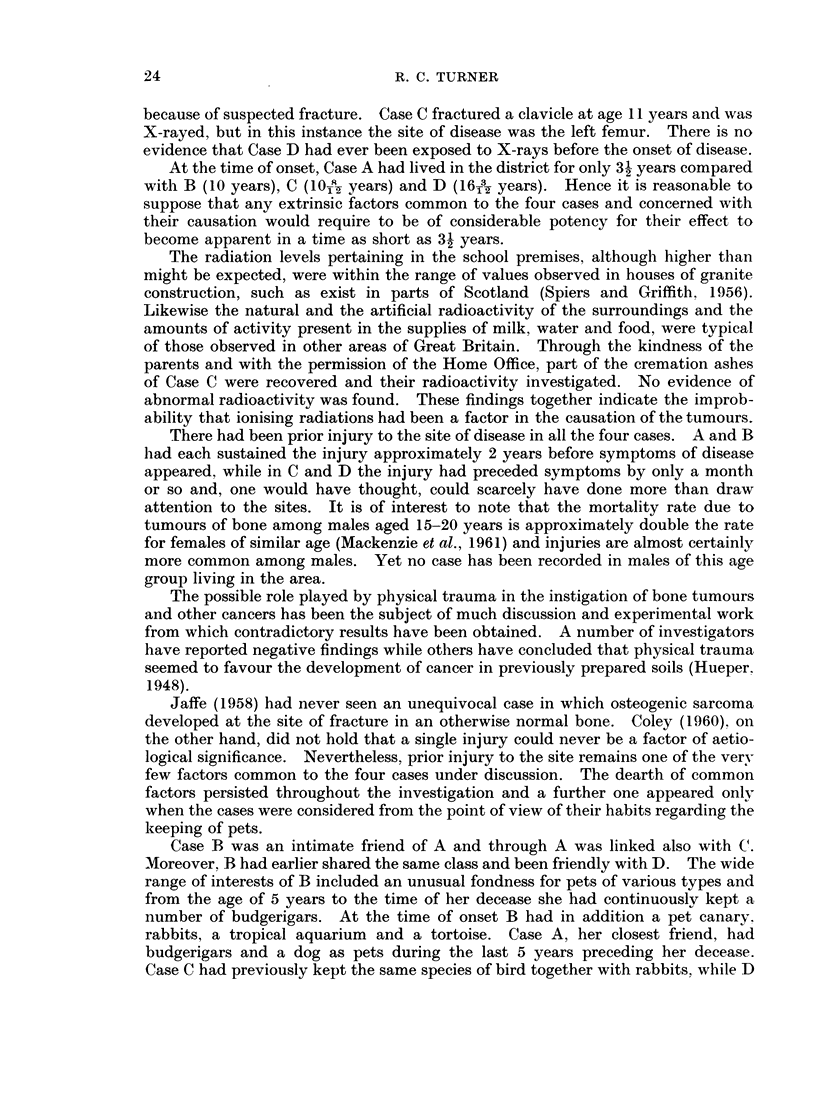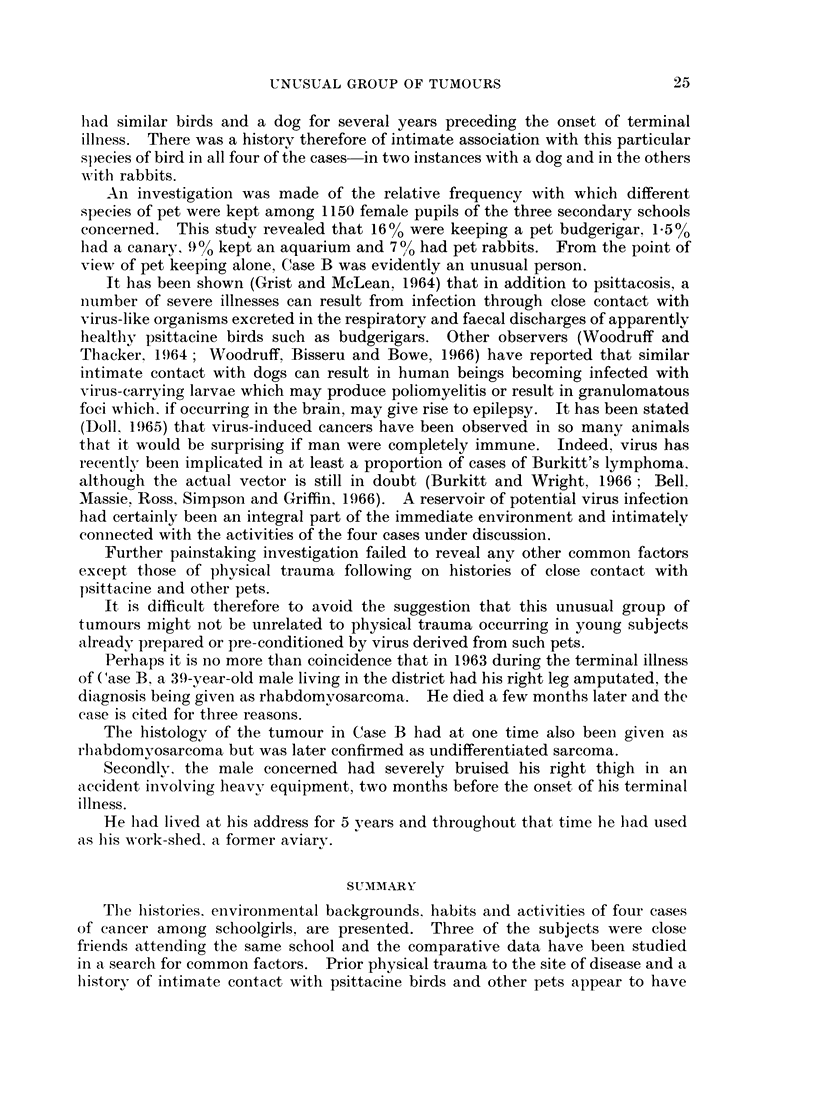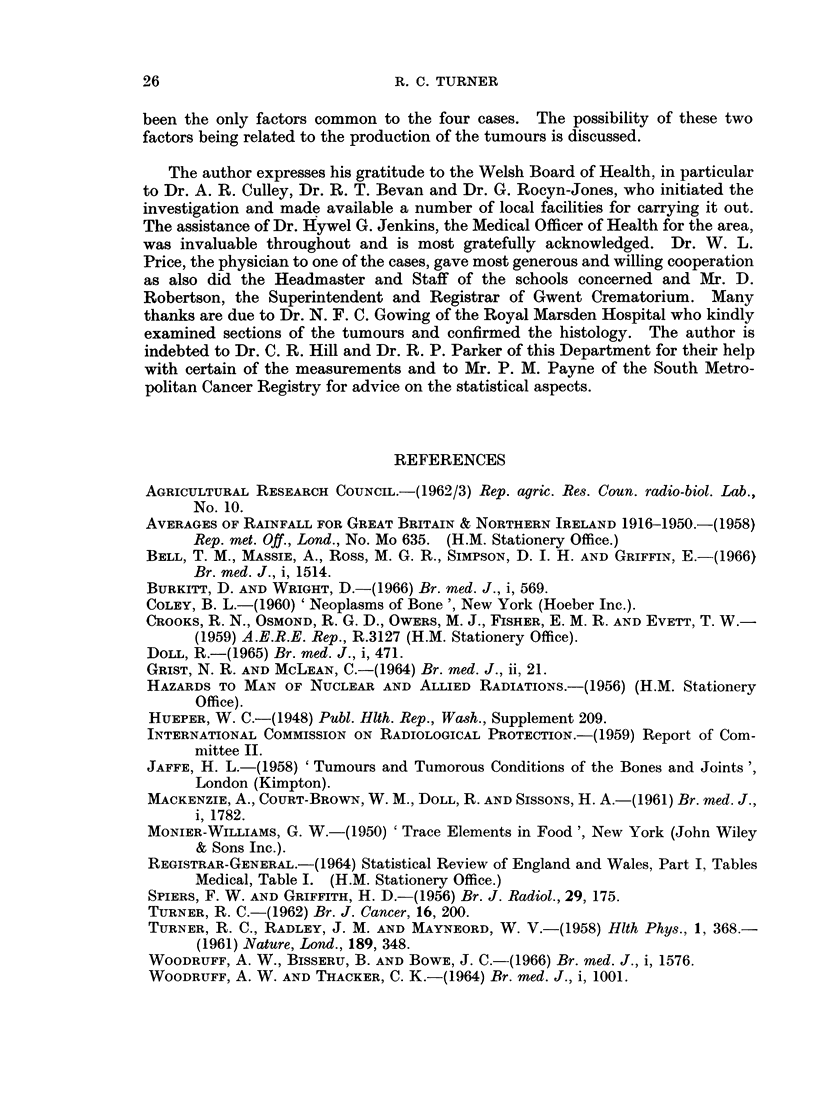# Unusual group of tumours among schoolgirls.

**DOI:** 10.1038/bjc.1967.3

**Published:** 1967-03

**Authors:** R. C. Turner


### UNUSUAL GROUP OF TUMOURS AMONG SCHOOLGIRLS

R. C. TURNER

*From the Department of Physics, Institute of Cancer Research, Royal Cancer Hospital, Fulham Road, London S.W.3*

Received for publication September 9, 1966

THIS paper records a group of four tumours, all rapidly fatal, which occurred among schoolgirls in a Monmouthshire town during the period January, 1961 to June, 1962. Two of the cases were primary tumours of bone, the histology in the others having initially been given as synovioma and subsequently as undifferentiated sarcoma. The group is unusual because these types of cancer are comparatively rare among females of this age and furthermore, three of the cases were closely associated as friends and pupils attending the same school. The fourth case was a pupil at a different school in the neighbourhood but had earlier been a classmate and associated with one of the other three. In 1964, some months after the decease of the fourth subject and at the invitation of the Welsh Board of Health, investigations were commenced into the histories, environmental backgrounds, habits and activities of the cases in a search for factors common to them all. The following is a summary of the considerable data collected during the investigations.

CASE A

Born July, 1945 at Norwich.
Father of Welsh extraction (Monmouthshire).
Mother—English (Norwich).
An only child.
Had a number of pedoscopies as a child in Norwich.

| | | |
|---|---|---|
| 1957 | (aged 12 years) | —Moved to Cwmbran (Mon.) and commenced at Croesyceiliog Secondary Modern School. |
| 1958 | ( ,, 13 ,, ) | —Transferred to adjacent Grammar School in the same class as Case C. Injured right knee by falling down steps and a few months later, the same knee injured by hockey ball. |
| Jan., 1961 | ( ,, $15\frac{1}{2}$ ,, ) | —Complained of pain in right knee—X-rayed. |
| Feb., 1961 | ( ,, ,, ,, ) | —Biopsy performed followed by amputation of right leg. |
| Mar., 1962 | ( ,, $16\frac{8}{12}$ ,, ) | —Died at home. |

Diagnosis given as sarcoma of right tibia.
Histology subsequently confirmed as osteosarcoma of right tibia.

CASE B

Born June, 1945 at Bridgend (Glamorgan).
Father and mother both of Welsh extraction (Glamorgan).
One brother 2 years older—no sisters.

| | | |
|---|---|---|
| 1952 | (aged 7 years) | —Moved to Cwmbran (Mon.). |
| 1956 | ( ,, 11 ,, ) | —Commenced at Llantarnam Secondary Modern School in same class as Case D. |

1957 ( ,, 11+ ,, ) —Transferred to Croesyceiliog Secondary Modern School.
1957 ( ,, 12 ,, ) —Transferred to adjacent Grammar School.
1960 ( ,, 15 ,, ) —Suspected fracture of right wrist due to a fall at school—wrist X-rayed—no fracture found.
July, 1962 ( ,, $17\frac{1}{12}$ ,, ) —Complained of pain and swelling of right wrist.
Sep., 1962 ( ,, $17\frac{3}{12}$ ,, ) —Right wrist X-rayed.
Oct., 1962 ( ,, $17\frac{4}{12}$ ,, ) —Lump excised from right wrist.
Nov., 1963 ( ,, $18\frac{5}{12}$ ,, ) —Died in hospital.
Diagnosis given as synovioma of right wrist (no bone named).
Histology given as rhabdomyosarcoma and later as undifferentiated sarcoma.

CASE C

Born August, 1946 at Walsall (Staffs.).
Father and mother both English.
One sister 4 years older—no brothers.
1951 (aged 5 years) —Moved to Cwmbran (Mon.).
1957 ( ,, 11 ,, ) —Commenced at Croesyceiliog Secondary Modern School. Fractured a clavicle—X-rayed.
1958 ( ,, 12 ,, ) —Transferred to adjacent Grammar School, in same class as Case A.
1962 ( ,, 15+ ,, ) —Injured left knee by a fall at net-ball.
Apr., 1962 ( ,, ,, ,, ) —Left knee injured while dancing.
May, 1962 —Complained of pain in left knee. Massive radiotherapy to left knee. Left mid-thigh amputation performed.
Nov., 1962 ( ,, $16\frac{3}{12}$ ,, ) —Died at home.
Histology confirmed as osteosarcoma of left femur.

CASE D

Born December, 1945 at Porth (Glamorgan).
Father and mother both of Welsh extraction (Glamorgan).
Two older sisters, 1 younger sister, 1 younger brother.
1945 (aged 2 weeks) —Moved to Cwmbran (Mon.).
1956 ( ,, 11 years) —Commenced at Llantarnam Secondary Modern School in same class as Case B.
Feb., 1962 ( ,, $16\frac{3}{12}$ ,, ) —Left leg injured by net-ball stand falling on it.
Mar., 1962 ( ,, $16\frac{4}{12}$ ,, ) —Complained of pain and swelling of left hip.
June, 1962 ( ,, $16\frac{1}{2}$ ,, ) —Left hip X-rayed.
Dec., 1962 ( ,, 17 ,, ) —Died in hospital.
Diagnosis given as synovioma of left thigh, no bone being mentioned.
Histology confirmed as undifferentiated sarcoma.

*Family Histories*

The family histories of the four cases showed no congenital malformations or other defects.

The only cancer histories among the grandparents were :

Case A : Paternal grandfather d. Ca. stomach.
Case C : Maternal grandfather d. Ca. prostate.
Paternal grandmother d. lymphosarcoma.

Three grandparents had died of heart trouble, one with chronic bronchitis, one of hernia, the remaining eight were alive and well.

The mothers of Cases A and D had both suffered toxaemia of pregnancy but otherwise there had been no illness of the mothers nor had any special investigations or X-ray examinations been carried out on them either before or during pregnancy.

The medical histories of the cases themselves, before the onset of terminal illness, are summarised in Table I.

TABLE I

| | A | B | C | D |
|---|---|---|---|---|
| Mumps | 1948 (3 years) | No. | No. | 1950 (5 years) |
| Chicken pox | 1950 (5 ,, ) | 1950 (5 years) | 1948 (2 years) | 1949 (4 ,, ) |
| Measles | 1952 (7 ,, ) | 1947 (2 ,, ) | 1952 (6 ,, ) | 1948 (3 ,, ) |
| Pertussis | No. | 1947 (2 ,, ) | 1951 (5 ,, ) | No. |
| Scarlet fever | 1952 (7 ,, ) | No. | No. | 1946 (1 year) |
| Vaccination | 1945 (as baby) | 1961 (16 years) | No. | 1945 (as baby) |
| Polio immun. | 1957 (12 years) | 1960 (15 ,, ) | No. | 1958 (13 years) |
| B.C.G. | 1958 (13 ,, ) | 1960 (15 ,, ) | No. | 1958 (13 ,, ) |
| X-ray examinations before onset | Pedoscopies as child | R. wrist 1960 (15 years) | Clavicle 1957 (11 years) | None. |

With the above exceptions, all four cases were regarded by parents and private doctors as being fit and healthy girls who had needed only minimal medical attention before onset of terminal illness.

### *Home Environment*

Three of the cases (A, B and C) were from comfortable homes in which there would be no dietary deficiencies or limitations due to economic pressure. Case D, on the other hand, was one of five children in a family less well situated financially and could well have been subject to dietary deficiencies. For a number of years preceding onset, case B had disliked milk, bread and tea and had drunk mostly coffee. Case D was very fond of all shellfish and ate them at every opportunity.

There was otherwise no evidence of dietary fads or predilection for particular foods or drinks in any of the cases.

### *Friends of the Cases*

The cases were associated as follows :

A, B and Y were inseparable friends and had been so for several years.
(Y is alive and well).
A and C were close friends and classmates.
C and X were close friends as also were B and X.
(X, the cousin of B, is alive and well).
B and D had been classmates and friends in 1956.

### *Personalities of the Cases*

Teachers and friends were unanimous in their opinions that all four girls were of above average intelligence, academically highly proficient at school and in the case of B, C and D were also outstanding at athletics and other organised sports and games. The following brief summaries were derived from the same sources.

*Case A*.—A shy, reserved person not over-fond of organised social activities or games. Although a member of the Girl Guides, she took little part in their activities and preferred cycling, walking and cookery. Most holidays were spent in Norwich—her birthplace. Very fond of pets and had kept birds and a dog for a number of years.

*Case B*.—A very energetic and enthusiastic person with a wide range of interests and keen on all sports and social affairs. Principal hobbies were Girl Guide activities, playing hockey and net-ball, walking, needlework, fashioning pewter and music. Unusually fond of pets and kept numbers of birds in addition to other animals. The oldest of the cases and from some points of view the central character.

*Case C*.—A vivacious person fond of company and keen on athletics, hurdling. net-ball, swimming and dancing. She had kept pet birds and rabbits before the onset of terminal illness.

*Case D*.—A person shy of social activities except for games. Principal hobbies were athletics, net-ball (School Captain), hockey and dressmaking. Kept pet birds and a dog.

### *Schools*

Cases A, B and C all spent part of their first year's secondary education at the Modern School in Croesyceiliog before being transferred to the immediately adjacent Grammar School. At the time of onset, they had been pupils at the latter school for $3\frac{8}{12}$, $6\frac{5}{12}$ and $4\frac{3}{12}$ years respectively. Case D had for 6 years been a pupil at Llantarnam Secondary Modern School (about 1 mile distant) when symptoms first appeared. All three schools are of similar modern design, constructed within the last 10 years and surrounded by extensive grounds and playing fields.

The radiation backgrounds throughout the entire premises and surroundings of all three schools were investigated systematically, special attention being paid to all areas used exclusively by female pupils. Bulk samples of the drinking water. milk and food supplies of the schools were obtained and their radioactivity and trace element contents examined.

### (a) *Radiation background*

The general background inside each of the buildings was found to be 2–3 times higher than expected—this being especially so in toilet rooms where " terrazzo "

like material had been used extensively for floors, walls, etc. No evidence of localised " hot spots " of radiation or of the presence of radioactive material was found anywhere within the buildings.

Increased levels of beta/gamma radiation were observed everywhere close to the grass surfaces of the surrounding campus in each case and especially in proximity to moss growing between paving stones. Samples were removed for examination and the results compared directly with those found in similar specimens growing in Surrey. Gamma-ray spectrometry indicated the presence of cerium 144, praseodymium 144, antimony 125, ruthenium 106, rhodium 106, caesium 137 and manganese 54 in both the Surrey and the school samples. The most intense peaks observed in each spectrum were those due to cerium 144 and caesium 137 and in both cases the spectrum was typical of that due to mixed fission products of age 2–3 years.

The gamma activities due to caesium 137 were :

School samples—50 micromicrocuries per gram moss.
Surrey ,, —29 ,, ,, ,, ,,

Hence the caesium 137 content of the school specimens was approximately 1·7 times greater than that in the Surrey samples.

It is worthy of note that the average annual rainfall in the Monmouthshire area is 44″ compared with 25·2″ in Surrey—also a ratio of approximately 1·7 times (Averages of Rainfall, 1958). The total beta activity of the school moss was 1600 micromicrocuries per gram, the strontium 90 content being 28 micromicrocuries per gram compared with 16·5 micromicrocuries per gram in the Surrey specimens—again a ratio of 1·7 times due to the higher rainfall in the Welsh area.

(b) *Drinking waters*

*Radon* 222.—The amounts of dissolved radon gas in the school drinking waters were less than 5 micromicrocuries per litre compared with the levels of 100–200 micromicrocuries per litre in drinking waters of the South East Region (Turner, Radley and Mayneord, 1961).

*Radium and daughters.*—The long lived alpha radioactivity due to the presence of radium 226 and its daughters was less than 0·5 micromicrocuries per litre. The corresponding figure for drinking water in the London area is approximately 0·7 micromicrocuries per litre (Turner, Radley and Mayneord, 1961).

*Thorium and daughters.*—No alpha activity due to thorium and its daughters was detected and the total activity due to these radioelements is therefore regarded as being less than 0·1 micromicrocuries per litre.

*Artificial radioactivity.*—The values were :

Strontium 90—1·52 micromicrocuries per litre.
Caesium 137 —0·11 ,, ,, ,,

These contents are typical of those observed in the higher rainfall areas of the country (Crooks *et al.*, 1959).

*Stable trace elements.*—The average values expressed in milligrams per litre were found to be :

| | | | |
|---|---|---|---|
| Ca | 26·5 | Sr | 0·14 |
| Mg | 4·5 | Mn | 0·04 |
| K | 0·5 | Ag | <0·1 |
| Cu | 0·5 | Fe | <0·1 |
| Pb | 0·24 | Ni | <0·1 |
| Zn | 0·24 | Cr | <0·1 |

(c) *Milk*

The characteristics of the milk supplied to the schools were compared directly with those of similar quantities of milk purchased in Surrey.

(i) *Radium and daughters.*—The long lived alpha activities due to these radioelements were as follows :

School milk—1·20 micromicrocuries per pint.
Surrey ,, —0·73 ,, ,, ,,

Both values are within the range 0·45–1·70 micromicrocuries per pint reported for 30 representative samples of cow milks in Great Britain (Turner, Radley and Mayneord, 1958).

(ii) *Potassium* 40.—Gamma spectroscopy confirmed that there was no difference between the radioactive potassium 40 contents of the milks from the two areas.

(iii) *Artificial radioactivity.*—The gamma activity due to caesium 137 was approximately 1·77 times higher in the school milk than in the Surrey equivalent. This is again a reflection of the relative rainfall in the two areas. In view of this finding and of the parallel trend exhibited by the amounts of strontium 90 and caesium 137 in milks sampled on a national scale, comparative measurements of the strontium 90 contents were not made (Agricultural Research Council, 1962/3).

(iv) *Stable trace elements.*—Within the limits of experimental error, no difference was observed between the amounts of calcium, iron, potassium, magnesium and strontium in the two specimens.

The figures for zinc contents were :

School milks—2·8 milligrams per pint.
Surrey ,, —2·0 ,, ,, ,,

These figures correspond to 4·9 and 3·6 parts per million respectively and are within the reported range of 2–8 p.p.m. (Monier-Williams, 1950).

Lead, copper, nickel, chromium and silver were not detected in either sample.

(d) *School meals*

(i) *Radium and thorium.*—The average intake of long lived alpha activity due to the presence of these radioelements in food is given as approximately 14·0 micromicrocuries per person per day (Turner, 1962). The average activity of each school meal was observed to be 1·5 micromicrocuries, well within the range of values contributed by any one meal.

(ii) *Potassium* 40.—The average value of the stable potassium content of each meal was 0·75 gram. Among the general population the daily intake of this element is regarded as approximately 3·0 grams per person per day (I.C.R.P., 1959). The potassium 40 contents of the school meals were therefore normal.

(iii) *Artificial radioactivity.*—The average ratio of caesium 137 to potassium 40 was found to be 0·1 so that each specimen meal contained approximately 9 micromicrocuries of gamma radioactivity due to caesium 137, compared with 90 micromicrocuries of similar activity due to naturally occurring potassium 40.

### DISCUSSION

The number of females aged between 15 and 20 years in the population of England and Wales during the period 1960–62 was approximately 1·813 million, *i.e.* $\sim$3·8% of the total population of males and females (Registrar-General, 1964).

The population of Cwmbran New Town in 1964 was estimated at 31,000 and if we assume that the age distribution was similar to that in the country as a whole, there would have been approximately 1200 females between the ages of 15 and 20 years in that population.

Females in this age group have a mortality rate due to primary tumours of bone (all bones included) of 7·61 per million per year, *i.e.* $9{\cdot}13 \times 10^{-3}$ deaths per year among 1200 such females (Mackenzie, Court-Brown, Doll and Sissons, 1961). Cases A and C, with osteosarcoma of tibia and femur respectively, were in this category and died within a month of each other in 1962.

The mortality rate due to tumours of connective tissue among females of this age group is lower and less clearly defined. During the three years 1960–62 there were 8 deaths from this cause compared with 48 deaths due to primary tumours of bone, among females aged 15–20 years (Registrar-General, 1964). The mortality rate due to this type of tumour may therefore be regarded as approximately 1·5 per million per year, *i.e.* $1{\cdot}8 \times 10^{-3}$ deaths per year in a group of 1200 such females. Cases B and D are included in this category since the diagnosis of undifferentiated sarcoma made no mention of bone. All four of the cases occurred within a period of 2 years. The probability of two cases of each type of tumour occurring by chance in two years in any group of 1200 females aged 15–20 years is evidently small and has been assessed at $p < 1 \times 10^{-5}$. This small probability demanded that investigation be made into the possibility that a common extrinsic factor might have been concerned with their causation.

Despite intensive study of the comparative data there have emerged surprisingly few factors common to all the four cases.

Both parents of B and of D were of Welsh extraction as also was the father of A. On the other hand, both parents of C were English as was the mother of A. It would be difficult therefore to argue that a common genetic factor of Welsh origin was involved.

The absence of congenital defects and the relatively few cancers among the immediate ancestors offers no clue, neither do the pre-natal or the early childhood histories of the cases themselves. In three only of the cases (A, B, C) was there any known exposure to X-rays before onset of terminal illness. It is not possible to estimate the number of exposures or the total radiation dose received by the lower limbs of Case A during the shoe-fitting examinations she underwent as a child. Such procedures have been condemned as likely to be dangerous to children (*Hazards to Man of Nuclear and Allied Radiations*, 1956). The right wrist of Case B (the site of subsequent disease) was X-rayed at age 15 years because of suspected fracture. Case C fractured a clavicle at age 11 years and was X-rayed, but in this instance the site of disease was the left femur. There is no evidence that Case D had ever been exposed to X-rays before the onset of disease.

At the time of onset, Case A had lived in the district for only $3\frac{1}{2}$ years compared with B (10 years), C ($10\frac{8}{12}$ years) and D ($16\frac{3}{12}$ years). Hence it is reasonable to suppose that any extrinsic factors common to the four cases and concerned with their causation would require to be of considerable potency for their effect to become apparent in a time as short as $3\frac{1}{2}$ years.

The radiation levels pertaining in the school premises, although higher than might be expected, were within the range of values observed in houses of granite construction, such as exist in parts of Scotland (Spiers and Griffith, 1956). Likewise the natural and the artificial radioactivity of the surroundings and the amounts of activity present in the supplies of milk, water and food, were typical of those observed in other areas of Great Britain. Through the kindness of the parents and with the permission of the Home Office, part of the cremation ashes of Case C were recovered and their radioactivity investigated. No evidence of abnormal radioactivity was found. These findings together indicate the improbability that ionising radiations had been a factor in the causation of the tumours.

There had been prior injury to the site of disease in all the four cases. A and B had each sustained the injury approximately 2 years before symptoms of disease appeared, while in C and D the injury had preceded symptoms by only a month or so and, one would have thought, could scarcely have done more than draw attention to the sites. It is of interest to note that the mortality rate due to tumours of bone among males aged 15–20 years is approximately double the rate for females of similar age (Mackenzie *et al.*, 1961) and injuries are almost certainly more common among males. Yet no case has been recorded in males of this age group living in the area.

The possible role played by physical trauma in the instigation of bone tumours and other cancers has been the subject of much discussion and experimental work from which contradictory results have been obtained. A number of investigators have reported negative findings while others have concluded that physical trauma seemed to favour the development of cancer in previously prepared soils (Hueper, 1948).

Jaffe (1958) had never seen an unequivocal case in which osteogenic sarcoma developed at the site of fracture in an otherwise normal bone. Coley (1960), on the other hand, did not hold that a single injury could never be a factor of aetiological significance. Nevertheless, prior injury to the site remains one of the very few factors common to the four cases under discussion. The dearth of common factors persisted throughout the investigation and a further one appeared only when the cases were considered from the point of view of their habits regarding the keeping of pets.

Case B was an intimate friend of A and through A was linked also with C. Moreover, B had earlier shared the same class and been friendly with D. The wide range of interests of B included an unusual fondness for pets of various types and from the age of 5 years to the time of her decease she had continuously kept a number of budgerigars. At the time of onset B had in addition a pet canary, rabbits, a tropical aquarium and a tortoise. Case A, her closest friend, had budgerigars and a dog as pets during the last 5 years preceding her decease. Case C had previously kept the same species of bird together with rabbits, while D

had similar birds and a dog for several years preceding the onset of terminal illness. There was a history therefore of intimate association with this particular species of bird in all four of the cases—in two instances with a dog and in the others with rabbits.

An investigation was made of the relative frequency with which different species of pet were kept among 1150 female pupils of the three secondary schools concerned. This study revealed that 16% were keeping a pet budgerigar, 1·5% had a canary, 9% kept an aquarium and 7% had pet rabbits. From the point of view of pet keeping alone, Case B was evidently an unusual person.

It has been shown (Grist and McLean, 1964) that in addition to psittacosis, a number of severe illnesses can result from infection through close contact with virus-like organisms excreted in the respiratory and faecal discharges of apparently healthy psittacine birds such as budgerigars. Other observers (Woodruff and Thacker, 1964; Woodruff, Bisseru and Bowe, 1966) have reported that similar intimate contact with dogs can result in human beings becoming infected with virus-carrying larvae which may produce poliomyelitis or result in granulomatous foci which, if occurring in the brain, may give rise to epilepsy. It has been stated (Doll, 1965) that virus-induced cancers have been observed in so many animals that it would be surprising if man were completely immune. Indeed, virus has recently been implicated in at least a proportion of cases of Burkitt's lymphoma, although the actual vector is still in doubt (Burkitt and Wright, 1966; Bell, Massie, Ross, Simpson and Griffin, 1966). A reservoir of potential virus infection had certainly been an integral part of the immediate environment and intimately connected with the activities of the four cases under discussion.

Further painstaking investigation failed to reveal any other common factors except those of physical trauma following on histories of close contact with psittacine and other pets.

It is difficult therefore to avoid the suggestion that this unusual group of tumours might not be unrelated to physical trauma occurring in young subjects already prepared or pre-conditioned by virus derived from such pets.

Perhaps it is no more than coincidence that in 1963 during the terminal illness of Case B, a 39-year-old male living in the district had his right leg amputated, the diagnosis being given as rhabdomyosarcoma. He died a few months later and the case is cited for three reasons.

The histology of the tumour in Case B had at one time also been given as rhabdomyosarcoma but was later confirmed as undifferentiated sarcoma.

Secondly, the male concerned had severely bruised his right thigh in an accident involving heavy equipment, two months before the onset of his terminal illness.

He had lived at his address for 5 years and throughout that time he had used as his work-shed, a former aviary.

### SUMMARY

The histories, environmental backgrounds, habits and activities of four cases of cancer among schoolgirls, are presented. Three of the subjects were close friends attending the same school and the comparative data have been studied in a search for common factors. Prior physical trauma to the site of disease and a history of intimate contact with psittacine birds and other pets appear to have been the only factors common to the four cases. The possibility of these two factors being related to the production of the tumours is discussed.

The author expresses his gratitude to the Welsh Board of Health, in particular to Dr. A. R. Culley, Dr. R. T. Bevan and Dr. G. Rocyn-Jones, who initiated the investigation and made available a number of local facilities for carrying it out. The assistance of Dr. Hywel G. Jenkins, the Medical Officer of Health for the area, was invaluable throughout and is most gratefully acknowledged. Dr. W. L. Price, the physician to one of the cases, gave most generous and willing cooperation as also did the Headmaster and Staff of the schools concerned and Mr. D. Robertson, the Superintendent and Registrar of Gwent Crematorium. Many thanks are due to Dr. N. F. C. Gowing of the Royal Marsden Hospital who kindly examined sections of the tumours and confirmed the histology. The author is indebted to Dr. C. R. Hill and Dr. R. P. Parker of this Department for their help with certain of the measurements and to Mr. P. M. Payne of the South Metropolitan Cancer Registry for advice on the statistical aspects.

### REFERENCES

AGRICULTURAL RESEARCH COUNCIL.—(1962/3) *Rep. agric. Res. Coun. radio-biol. Lab.*, No. 10.

AVERAGES OF RAINFALL FOR GREAT BRITAIN & NORTHERN IRELAND 1916–1950.—(1958) *Rep. met. Off., Lond.*, No. Mo 635. (H.M. Stationery Office.)

BELL, T. M., MASSIE, A., ROSS, M. G. R., SIMPSON, D. I. H. AND GRIFFIN, E.—(1966) *Br. med. J.*, i, 1514.

BURKITT, D. AND WRIGHT, D.—(1966) *Br. med. J.*, i, 569.

COLEY, B. L.—(1960) ' Neoplasms of Bone ', New York (Hoeber Inc.).

CROOKS, R. N., OSMOND, R. G. D., OWERS, M. J., FISHER, E. M. R. AND EVETT, T. W.—(1959) *A.E.R.E. Rep.*, R.3127 (H.M. Stationery Office).

DOLL, R.—(1965) *Br. med. J.*, i, 471.

GRIST, N. R. AND McLEAN, C.—(1964) *Br. med. J.*, ii, 21.

HAZARDS TO MAN OF NUCLEAR AND ALLIED RADIATIONS.—(1956) (H.M. Stationery Office).

HUEPER, W. C.—(1948) *Publ. Hlth. Rep., Wash.*, Supplement 209.

INTERNATIONAL COMMISSION ON RADIOLOGICAL PROTECTION.—(1959) Report of Committee II.

JAFFE, H. L.—(1958) ' Tumours and Tumorous Conditions of the Bones and Joints ', London (Kimpton).

MACKENZIE, A., COURT-BROWN, W. M., DOLL, R. AND SISSONS, H. A.—(1961) *Br. med. J.*, i, 1782.

MONIER-WILLIAMS, G. W.—(1950) ' Trace Elements in Food ', New York (John Wiley & Sons Inc.).

REGISTRAR-GENERAL.—(1964) Statistical Review of England and Wales, Part I, Tables Medical, Table I. (H.M. Stationery Office.)

SPIERS, F. W. AND GRIFFITH, H. D.—(1956) *Br. J. Radiol.*, **29**, 175.

TURNER, R. C.—(1962) *Br. J. Cancer*, **16**, 200.

TURNER, R. C., RADLEY, J. M. AND MAYNEORD, W. V.—(1958) *Hlth Phys.*, **1**, 368.—(1961) *Nature, Lond.*, **189**, 348.

WOODRUFF, A. W., BISSERU, B. AND BOWE, J. C.—(1966) *Br. med. J.*, i, 1576.

WOODRUFF, A. W. AND THACKER, C. K.—(1964) *Br. med. J.*, i, 1001.